# Two-step widefield fundus fluorescein angiography-assisted laser photocoagulation in pediatric retinal vasculopathy: A pilot study

**DOI:** 10.3389/fmed.2022.961152

**Published:** 2022-08-23

**Authors:** Jie Peng, Jianing Ren, Xuerui Zhang, Yuan Yang, Yihua Zou, Haodong Xiao, Yu Xu, Peiquan Zhao

**Affiliations:** Department of Ophthalmology, Xin Hua Hospital Affiliated to Shanghai Jiao Tong University School of Medicine, Shanghai, China

**Keywords:** fundus fluorescein angiography (FFA), retinal vasculopathy, neovascularization, laser photocoagulation, fluorescein leakage

## Abstract

**Purpose:**

To introduce the procedures of two-step fundus fluorescein angiography (FFA) and evaluate its utility in the management of pediatric retinal vasculopathy.

**Materials and methods:**

In this retrospective study, medical records of 12 patients who received two-step FFA were studied. The two-step FFA consisted of step 1 [low-dose (LD)] FFA at an intravenous dose of 1.5 mg/kg fluorescein, followed by step 2 [reduced dose (RD)] FFA at a dose of 6.2 mg/kg fluorescein. Demographic data, including age, gender, diagnosis, weight, gestational age, birth weight, and weight on the examination day were taken, were collected. The results of two-step FFA and treatment were recorded.

**Results:**

A total of 20 eyes were studied. The top 5 common FFA changes in RD-FFA included peripheral avascular zone (15 eyes), fluorescein leakage (10 eyes), supernumerous vascular branching (10 eyes), neovascularization (NV) (8 eyes), and absence of the foveal avascular zone (6 eyes). LD-FFA was efficient to show all the NV without severe vitreous dye in 8/8 (100.0%) eyes with NV, partial peripheral avascular zone in 11/15 (73.3%) eyes, while RD-FFA always offered more information in all the eyes. Thirteen eyes had laser photocoagulation under the guidance of LD-FFA. In 4 (30.8%) eyes, RD-FFA revealed more lesions and an immediate relaser was performed. Laser photocoagulation was successfully performed in all the 13 eyes in one session without being rearranged. After a median follow-up of 28.1 months, all the eyes were in a stable status.

**Conclusion:**

Step-one LD-FFA acted as a pre-FFA to show the NV, and step-two RD-FFA acted as a double-check. The modified strategy may be a helpful clinical adjuvant in the laser photocoagulation of pediatric retinal disorders, especially for young ophthalmologists.

## Introduction

Widefield fundus fluorescein angiography (WF-FFA) offers more details of microvascular abnormalities, retinal non-perfusion areas, neovascularization, vascular fluorescein leakage (1), and vascular perfusion dynamics than fundoscopy or color image alone, broadening ophthalmologists’ understanding of pediatric retinal vascular disorders (2). Fundus fluorescein angiography (FFA) also assisted in the management of pediatric retinal vascular diseases such as retinopathy of prematurity (ROP) (3), Coats disease, familial exudative vitreoretinopathy (FEVR) (4), incontinentia pigmenti (IP) (5), and so on.

Traditional FFA was performed with an intravenous injection of 5% fluorescein dye dosed by weight (7.7 mg/kg) (6, 7). For oral FFA, the applied concentration of fluorescein was 20 mg/kg of body weight as reported (8, 9). Sometimes, the green fluorescein dye extravasated into the vitreous overlying the areas of retinal neovascularization (NV) guiding the ablation of avascular retina (10, 11). Unfortunately, for cases with massive NV, extensive leakage of fluorescein into the vitreous made laser photocoagulation difficult. The treatment should be rescheduled (12), which always needs another general anesthesia for younger children.

Occasionally, we found 1.5 mg/kg fluorescein that could help to show the retinal NV without severe vitreous dye. To avoid secondary interference caused by dye leakage, we came up with this two-step FFA procedure and evaluated its utilization in a real-world setting. The two-step FFA consisted of step 1 [low-dose (LD)] FFA at an intravenous dose of 1.5 mg/kg fluorescein, followed by step 2 (reduced dose of 6.2 mg/kg) FFA to ensure the observation and treatment in pediatric patients. In this pilot study, we postulated that two-step FFA may be increasing as a meaningful clinical adjuvant in the management of pediatric retinal disorders.

## Materials and methods

With Institutional Review Board approval by Xinhua Hospital Affiliated to Shanghai Jiao Tong University (XHEC-D-2022-071), a retrospective study was conducted in our department from 6 December 2019 to 15 May 2022. It adhered to the tenets of the Declaration of Helsinki (13). Written informed consent was signed by the guardians for performing FFA and expected interventions, including laser photocoagulation or intravitreal injection of anti-vascular endothelial growth factor (VEGF) agents. Cases were excluded if poor-quality images were acquired due to significant media opacity such as keratopathy, cataract, or vitreous hemorrhage.

Demographic data, including age, gender, diagnosis, weight, gestational age, birth weight, and weight on the examination day were collected. All the FFA and fundus color images were acquired under general anesthesia with RetCam III (Clarity Medical System, Pleasanton, CA, United States). Scleral indentation was performed as needed to show the far periphery of the retina.

After proper general anesthesia and mydriasis, color images were initially taken before step 1 FFA (LD-FFA). Intravenous sodium fluorescein (10%) was injected into the peripheral vein followed by a saline flush. Contact high-resolution WF-FFA images were obtained. To avoid possible neglect of lesions of both eyes, speculums were placed on both eyes simultaneously. FFA images of posterior pole and four quadrants were first taken on the eye with severer abnormalities or possible NV and the camera was shifted to the fellow eye quickly to take the early venous-stage images. After the LD-FFA, we also checked the skin and vital signs for any allergic reactions. Only when no severe allergic reactions occurred, the further procedures performed. LD-FFA-guided laser photocoagulation was carried out wherever required immediately. After laser photocoagulation, step 2 FFA with a dose of 6.2 mg/kg sodium fluorescein FFA [reduced dose FFA (RD-FFA)] was performed to confirm the possible residual lesions and proper laser spots. Relaser treatment can be applied to the skipped areas accordingly. For eyes with massive NV or severe fluorescein leakage, intravitreal injection of ranibizumab could be applied after the laser photocoagulation.

### Statistics

Statistical analysis was performed using Statistical Package for the Social Sciences (SPSS) version 26 (IBM Corporation, Armonk, NY, United States). Data were expressed as the mean ± SD or as median and range.

## Results

Twelve patients (6 boys and 6 girls) with a median age of 6.70 (4.63, 47.75) months, and a mean birth weight of 3070.83 ± 751.50 g received two-step FFA were studied. The clinical diagnoses included FEVR (6 cases, 50.0%), IP (3 cases, 25.0%), Coats disease (2 cases, 16.7%), and retinal detachment (RD) with unknown reason (1 case, 8.3%). Eight patients underwent bilateral FFA and four patients underwent unilateral FFA due to simultaneous surgery on the fellow eye. As a result, a total of 20 eyes were studied.

**FIGURE 1:**
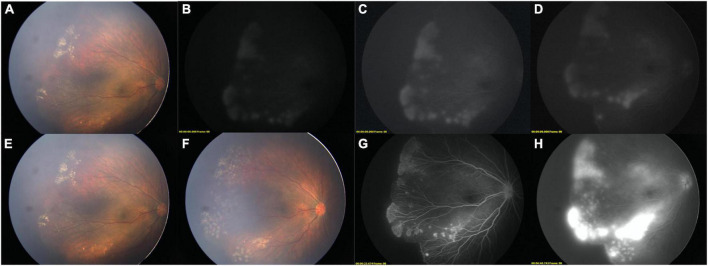
Two-step FFA of a FEVR child. **(A)** Color image before FFA (OD). **(B,C)** [adjusted version of **(B)**], LD-FFA image showed supernumerous vascular branching, NV, fluorescein leakage, peripheral avascular zone. **(D)** adjusted version of late phase of LD-FFA, fluorescein leakage remained. **(E)** Color image after LD-FFA. Comparing with **(A)**, no obvious media opacity was noted. **(F)** After laser photocoagulation, the laser spots and retinal vessels are clearly displaced. **(G)** Step-two RD-FFA shown clearly all the detailed retinal vessels and revealed more about the absence of the FAZ, and bulbous vascular terminals. **(H)** Late phase of RD-FFA, severe fluorescein leakage was noted.

**FIGURE 2:**
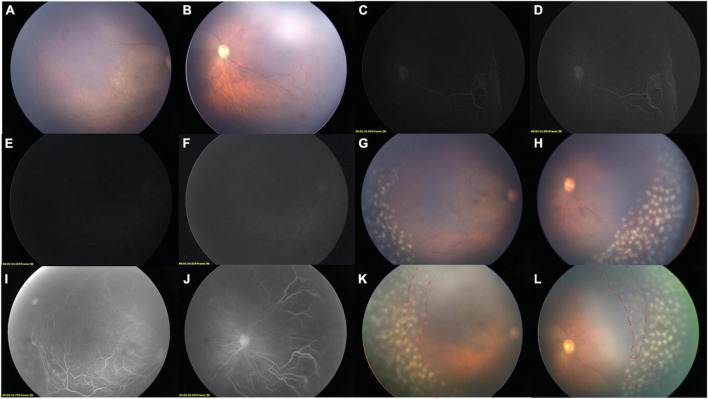
Two-step FFA-guided relaser of an IP child. Color images of the right eye before FFA [**(A)** Temporal field; **(B)** Nasal Field]. LD-FFA [**(C)**: 1 m 35 s, **(D)**: Adjusted version of **(C)**; **(E)**: 1 m 54 s, **(F)** adjusted version of **(E)**] showed supernumerous vascular branching, vessels dilatation, vessels tortuosity, and peripheral avascular zone in the nasal field **(D)**, while not so clear in the temporal side **(E,F)**. **(F)** Showed filling of optic disk and sparse posterior retinal vessels. **(G,H)** Color images after laser ablations, the media was clean enough without green stain by the fluorescein. **(I,J)** Step-two RD-FFA reveals all the abnormal vessels, non-perfusion areas, and previous laser spots. **(K,L)** After comparisons, supplementary laser photocoagulation was immediately applied (red dashed line), while the vitreous is greenish due to dye extravasation after RD-FFA.

**FIGURE 3:**
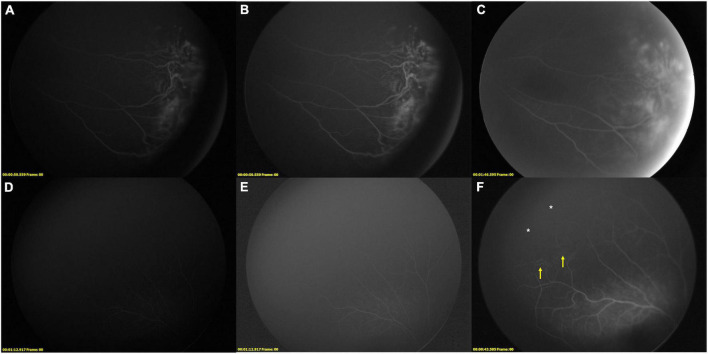
Supplementary relaser in the fellow eye after RD-FFA. **(A–F)** FFA images of case 10, a 5-year-old girl with IP. **(A,B)** [**(B)**: adjusted version of **(A)**], LD-FFA (OS): showed dragged-disk, vessels dilatation, vessels tortuosity, messy vessels with fluorescein leakage, and peripheral avascular zone in the temporal field. C, RD-FFA (OS) showed dragged-disk, vessels dilatation, vessels tortuosity, messy vessels, fluorescein leakage, telangiectasis, peripheral avascular zone, and laser spots in the temporal field. **(D,E)** [**(E)**: edited version of **(D)**], LD-FFA (OD) was obscure and showed sparse posterior vessels. **(F)** RD-FFA (OD) showed vessels tortuosity and messy vessels in the periphery (yellow arrow) and peripheral avascular zone (white asterisk) in the superotemporal field.

The results are given in Table 1 and shown in Figures 1, 2. Detailed results are shown in Supplementary material. In LD-FFA, due to the low dose, the early stage images are not clear, and it is hard to illustrate accurate arm-retina time (ART) and timing of phases in all the cases. In LD-FFA, the images can be dark sometimes, with built-in software built in Retcam III, the adjusted images can offer more information in all the images (Figures 1C,D, 2D,F, 3B,E,F). The top 5 common FFA changes in RD-FFA included peripheral avascular zone (15 eyes, 75.0%), fluorescein leakage (10 eyes, 50.0%), supernumerous vascular branching (10 eyes, 50.0%), NV (8 eyes, 40.0%), absence of the foveal avascular zone (FAZ) (6 eyes, 30.0%), and vessels tortuosity (5 eyes, 25.0%). The top 5 common FFA changes in LD-FFA included peripheral avascular zone (11 eyes, 55.0%), fluorescein leakage (9 eyes, 45.0%), NV (8 eyes, 40.00%), supernumerous vascular branching (7 eyes, 35.0%), vessels dilatation (4 eyes, 20.0%), and vessels tortuosity (4 eyes, 20.0%). With a higher dose of fluorescein, RD-FFA reveals more lesions than LD-FFA (Table 1). Interestingly, some lesions were only shown in RD-FFA, including microaneurysms, vessels fluorescence staining, absence of the FAZ, bulbous vascular terminals, distinct pruning of vessels, persistent fetal vasculature (PFV), capillary dropout, and peripheral vessels in normal eyes.

**TABLE 1:** Results of the two-step FFA.

	Step one, RD-FFA, eye(s)	Step two, LD-FFA, eye(s)	RD-FFA shown more than LD-FFA, eye(s)
Peripheral avascular zone	15	11	4
Fluorescein leakage	10	9	1
Supernumerous vascular branching	10	7	3
Neovascularization	8	8	0
Absence of the FAZ	6	0	6
Vessels tortuosity	5	4	1
Bulbous vascular terminals	5	0	5
Normal	4	0	4
Dragged-disc	4	3	1
Vessels dilatation	4	4	0
Microaneurysms	3	0	3
Messy vessels	3	1	2
Old laser spots	2	2	0
Vessels fluorescence staining	2	0	2
Telangiectasia	3	2	1
Distinct pruning of vessels	2	0	2
Capillary dropout	2	0	2
Fine vessels	1	1	0
Retinal fold	1	1	0
Avascular zone in the posterior pole	1	1	0
Retinal detachment	1	1	0
Venous-venous anastomoses	1	1	0
Persistent fetal vasculature	1	0	1

FAZ, foveal avascular zone; LD-FFA, low dose-fundus fluorescein angiography; RD-FFA, reduced dose-fundus fluorescein angiography.

Low-dose fundus fluorescein angiography was efficient to show all the NV with fluorescein leakage in 8/8 (100.0%) eyes. In 14 eyes with different kinds of abnormal vessels, LD-FFA always showed less information and neglected some information in 14 (100%) eyes. Among 15 eyes with peripheral avascular zone, LD-FFA showed partial peripheral avascular zone in 11 eyes frontier to abnormal vessels or NV, while LD-FFA was unable to show peripheral avascular zone anterior to normal or fine vessels, and not efficient to show all the peripheral avascular zone in all the 15 eyes as shown in RD-FFA. As a double-edged sword, low-dose (1.5 mg/kg) fluorescein could show NV in LD-FFA without obvious disturbance in the color fundus images in all the 8/8 eyes. With a higher dose, RD-FFA shows more distinct vascular details about peripheral retinal anatomy, vascular network, and non-perfusion areas, and necessary clinical information was obtained in all the cases. As laser spots were performed after the LD-FFA, laser spots were not recorded as an FFA change.

In total, 13 (65.00%) eyes had laser photocoagulation under the guidance of LD-FFA. In 4 (4/13, 30.77%) eyes of them, RD-FFA revealed more lesions asking for immediate relaser (Figure 3). The reasons for the relaser included more abnormal vessels and non-perfusion areas (2 eyes) and abnormal vessels with local fluorescein leakage (2 eyes). Laser photocoagulation was successfully performed in all the 13 eyes in one session without being rearranged. Four eyes received intravitreal injection of ranibizumab after laser photocoagulation.

All the patients tolerated the procedures without adverse effects during or after the procedures, such as rash, respiratory distress, tachycardia, fever, and local injection site reactions as reported in adults (14). The median follow-up duration was 28.15 (25.78, 28.70) months. On the last visit, all the eyes were in a stable status. No progression of diseases, recurrence of vascular activity, or laser-induced complications, such as rhegmatogenous RD, retinal hole, retinal hemorrhage, exudative RD, or retinal holes, were observed.

## Discussion

Fundus fluorescein angiography is helpful in clinical situations for a variety of pediatric retinal diseases. In patients with darkly pigmented fundi, such as Asians, the border of the vascularized zone and the demarcation line are barely detectable with an ophthalmoscope. Intraoperative FFA-guided laser photocoagulation in children offered good anatomic outcomes, with preservation of visual acuity in most cases of retinal vascular disease (11, 12, 15). Green fluorescein dye extravasated into the vitreous overlying the areas of retinal neovascularization could guide ablation of the avascular retina (10, 11). However, as time goes by, the dye would diffuse to surrounding areas and stain the area that should not be ablated. Besides, when massive NVs and dye leakage occurred, the dye will be too severe to see the fundus, making simultaneous laser treatment difficult. The treatment should be rescheduled sometimes (12).

Fundus fluorescein angiography was commonly performed with intravenous injection of fluorescein at a dose of 7.7 mg/kg (6, 7) or 3.5 mg/kg (3) and oral intake of fluorescein at a dose of 20 mg/kg (8, 9). The total dose of two-step FFA was also 7.7 mg/kg, same as many other studies (2, 7), which is safely administered intravenously in patients of all the ages, including medically frail, preterm infants (16). The procedures were successfully performed with no fluorescein-related complications in this study. We just made a small modification to divide the common dose into two phases, which are technically safe as traditional ones.

The LD-FFA was able to show all the NV in all the eyes (100.00%), which is helpful in clinical applications. It showed the retinal NVs without strong vitreous staining, making it easy to evaluate the retinal vessels and laser spots under the indirect ophthalmoscope and the camera. A clear view is very important for the doctor to give precise treatment. Inappropriate laser photocoagulation may end up with disease progression, including rhegmatogenous RD, tractional RD, or a combination of both (17). With the guidance of LD-FFA, the NV and abnormal vessels were directed without obvious fluorescein leakage, making precise laser ablations easier to be performed.

For pediatric patients with poor compliance, the FFA and laser photocoagulation are always performed under general anesthesia in the spine position. The laser ablations are conducted with binocular laser indirect ophthalmoscope, which needs a longer learning curve. The procedures are even harder with obscure media. Step-one LD-FFA acted as a pre-FFA to show the NV without obvious vitreous opacity. As a double-edged sword, LD-FFA was surely unable to show all the vascular anomalies, especially for subtle lesions, such as microaneurysms, vessels fluorescence staining, absence of the FAZ, bulbous vascular terminals, distinct pruning of vessels, PFV, and peripheral vessels in normal eyes. Hence, RD-FFA can serve as a good complement and confirmation on this basis. Step-two RD-FFA reveals more detailed information about retinal vessels and new laser spots, which is the double-check and guides supplementary laser photocoagulation when needed. On the basis of former laser ablation, the residual supplementary laser photocoagulation required less time before excessive fluorescein extravasation blurred the view, which is friendly to surgeons with comfort, especially for young ophthalmologists in their early career.

As a result, in our opinion, we highly recommend this technique to be applied in cases with retinal exudates and suspected NV. An LD-FFA will show whether and where the NV existed without severe media opacity, making the laser treatment easier. With the same pharmacokinetics, the circulation time is the same as traditional ones. So, the NV is always shown within 1 min. If no NVs were noted 1 min after the fluorescein injection, the RD-FFA could be performed immediately to save time. The step-one LD-FFA could be performed as a pretest for NV.

Besides, lesions of the fellow eye could be sometimes neglected since the quality of the angiogram of the fellow eye was suboptimal when performed at a late stage of the angiogram sometimes (12). In case 10 with IP, subtle peripheral messy vessels and avascular zone were noted in RD-FFA and supplementary laser ablation was applied. We believe that an RD-FFA gives us another chance to possibly reveal more signs, which may be neglected in a one-time FFA. After a median follow-up duration of 28.15 months, no laser-induced complications were noted. Besides, all the 13 eyes that received laser photocoagulation were in a stable status without active anomalies. Two-step FFA-guided laser photocoagulation helped to take the active lesions under control.

Furthermore, the LD-FFA could also be considered an allergic test for fluorescein. After the LD-FFA, we also checked the skin and vital signs for any allergic reactions. Only when no severe allergic reactions occurred, the further procedures performed. The LD-FFA instead of a full dose may evoke less severe FFA-associated complications, which could also make the procedure a safer one.

With no doubt, there are drawbacks of this two-step FFA and limitations of the pilot study. First, the modified technique with two steps is more time-consuming than traditional ones, and with more risks for corneal or conjunctival abrasions. Besides, the doses for each step may not be the optimal ones. There was only one dose without a gradient method to test the most suitable dose strategies. The perfect dose should always be one effective enough to show all the abnormal vessels without severe vitreous dye, which is most optimal when personalized. In our opinion, due to disease heterogeneity, the ideal dose should be decided in multiple FFA in the same group of patients in different concentration gradients and choose the best one. However, it is impossible in a clinical set. Furthermore, with LD-FFA, the vessels are commonly obscure; it was harder to fetch good images with the right position and perfect focus, which may rely on an experienced photographer. Other limitations included those biases intrinsic to a retrospective study. The study sample was small, so it was only a pilot study. It was impossible to evaluate all the vascular lesions in this study. At last, ideally, a comparison group with traditional FFA with a regular dose should be arranged to evaluate this two-step FFA’s feasibility. However, in this pilot study, we merely want to introduce the concept and first attempts. A further study with a larger scale, gradient doses, and comparison groups is needed.

In summary, we postulated a modified strategy with two-step widefield FFA. The step-one FFA is effective to show retinal NV without severe fluorescein leakage. The step-two FFA could also be a double-check to prevent possible skip areas. The strategy may be a helpful clinical adjuvant in the laser photocoagulation of pediatric retinal disorders, especially for young ophthalmologists early in their careers in laser photocoagulation.

## Data availability statement

The original contributions presented in this study are included in the article/Supplementary material, further inquiries can be directed to the corresponding authors.

## Ethics statement

The studies involving human participants were reviewed and approved by the Institutional Review Board approval by Xinhua Hospital Affiliated to Shanghai Jiao Tong University. Written informed consent to participate in this study was provided by the participants’ legal guardian/next of kin.

## Author contributions

JP, JR, XZ, YY, YZ, and HX collected, analyzed, and interpreted the data. JP drafted the manuscript. JR, XZ, YY, YZ, and HX carefully revised the manuscript. YX and PZ contributed to the conception of the study. All authors have read and approved the final version of the manuscript.
