# Supplementary material for: Two-step widefield fundus fluorescein angiography-assisted laser photocoagulation in pediatric retinal vasculopathy: A pilot study
Source: Front Med (Lausanne). 2022 Aug 23;9:961152. doi: 10.3389/fmed.2022.961152 (PMC9445166; doi:10.3389/fmed.2022.961152)
Supplement: Supplementary file 1 [file Data_Sheet_1.pdf]

Detailed results of FFA

Step 1 Low-dose (LD)-FFA results:

1. peripheral avascular zone (11 eyes): 1OD; 2OD; 3OD; 5OD;5OS; 6OD; 8OD; 10OS; 11OD; 11OS; 12OD;
2. fluorescein leakage (9 eyes): 2OD; 4OS; 5OD;5OS; 6OD; 8OD; 10OS; 11OD; 11OS;
3. NV (8 eyes): 2OD; 4OS; 5OD;5OS; 6OD; 8OD; 11OD; 11OS;
4. supernumerous vascular branching (7eyes): 2OD; 3OD; 6OD;8OD; 9OS; 11OD; 11OS;
5. vessels dilatation (4 eyes): 1OD; 3OD; 10OS; 12OD;
6. vessels tortuosity (4 eyes): 1OD; 3OD; 9OS; 10OS;
7. dragged-disc (3 eyes): 4OS;6OD; 10OS;
8. Telangiectasia (2 eyes): 1OD; 12OD;
9. old laser spots (2 eyes): 11OD; 11OS;
10. avascular zone in the posterior pole (1 eye): 4OS;
11. retinal fold (1 eye): 4OS;
12. fine vessels (1 eye): 9OS;
13. retinal detachment (1 eye):6OS;
14. venous-venous anastomoses (1 eye): 8OD;
15. messy vessels (1 eye): 10OS;
16. microaneurysms (0 eyes):None;
17. vessels fluorescence staining (0 eyes): None;
18. Normal (0 eyes):None. Obscure images with sparse vessels;
19. absence of the FAZ (0 eyes): None;
20. bulbous vascular terminals (0 eyes):None;
21. distinct pruning of vessels (0 eye): None.
22. PFV (0 eye): None;
23. Capillary dropout(0 eyes):None.

Step 2 Reduced dose(RD)-FFA

1. peripheral avascular zone (15 eyes): 1OD; 2OD; 3OD;3OS; 4OS;5OD;5OS; 6OD; 8OD; 9OS; 10OD; 10OS; 11OD; 11OS; 12OD;
2. fluorescein leakage (10 eyes): 2OD; 4OS; 5OD;5OS; 6OD; 6OS; 8OD; 10OS; 11OD; 11OS;
3. supernumerous vascular branching (10 eyes): 2OD; 3OD; 4OS; 5OD;5OS; 6OD;8OD; 9OS; 11OD; 11OS;
4. NV (8 eyes): 2OD; 4OS; 5OD;5OS; 6OD; 8OD; 11OD; 11OS;
5. absence of the FAZ (6 eyes): 2OD; 3OD; 8OD; 11OD; 11OS.4OS;
6. vessels tortuosity (5 eyes): 1OD; 3OD; 9OS; 10OD; 10OS;
7. Normal (4 eyes):1OS; 7OD; 8OS; 12OS;
8. dragged-disc (4 eyes): 6OD; 10OS;11OS;4OS;
9. vessels dilatation (4 eyes): 1OD; 3OD; 10OS; 12OD;
10. microaneurysms (3 eyes): 1OD; 3OD; 12OD;
11. messy vessels (3 eyes): 8OD; 10OD; 10OS;
12. telangiectasia (3 eyes): 1OD; 12OD;10OS;
13.

14. old laser spots (2 eyes): 11OD; 11OS;
15. vessels fluorescence staining (2 eyes): 1OD; 3OD;
16. distinct pruning of vessels (2 eyes): 5OD;5OS;
17. fine vessels (1 eye): 9OS;
18. bulbous vascular terminals (5 eyes): 2OD; 4OS; 5OD;5OS; 6OD;
19. capillary dropout(2 eyes)1Od;12OD;
20. retinal fold (1 eye): 4OS;
21. avascular zone in the posterior pole (1 eye): 4OS;
22. retinal detachment (1 eye):6OS;
23. venous-venous anastomoses (1 eye): 8OD;
24. PFV (1 eyes): 11OS;

Detailed results of laser treatment

No Laser (7 eyes): 1OS; 3OS; 7OD;9OS;6OS;8OS;12OS;

Enough laser (9 eyes): 1OD; 2OD; 4OS;5OS; 6OD;8OD;10OS;11OD;11OS;

Re-laser((4 eyes)): 3OD; 5OD;10OD;12OD.
